# Tumor Environmental Factors Glucose Deprivation and Lactic Acidosis Induce Mitotic Chromosomal Instability – An Implication in Aneuploid Human Tumors

**DOI:** 10.1371/journal.pone.0063054

**Published:** 2013-05-10

**Authors:** Chunyan Dai, Feifei Sun, Chunpeng Zhu, Xun Hu

**Affiliations:** 1 Cancer Institute (a Key Laboratory for Cancer Intervention and Prevention of China National Ministry of Education), The Second Affiliated Hospital, Zhejiang University School of Medicine, Hangzhou, China; 2 State Key Laboratory of Oncology in South China, Sun Yat-sen University Cancer Center, Guangzhou, China; Duke University, United States of America

## Abstract

Mitotic chromosomal instability (CIN) plays important roles in tumor progression, but what causes CIN is incompletely understood. In general, tumor CIN arises from abnormal mitosis, which is caused by either intrinsic or extrinsic factors. While intrinsic factors such as mitotic checkpoint genes have been intensively studied, the impact of tumor microenvironmental factors on tumor CIN is largely unknown. We investigate if glucose deprivation and lactic acidosis – two tumor microenvironmental factors – could induce cancer cell CIN. We show that glucose deprivation with lactic acidosis significantly increases CIN in 4T1, MCF-7 and HCT116 scored by micronuclei, or aneuploidy, or abnormal mitosis, potentially via damaging DNA, up-regulating mitotic checkpoint genes, and/or amplifying centrosome. Of note, the feature of CIN induced by glucose deprivation with lactic acidosis is similar to that of aneuploid human tumors. We conclude that tumor environmental factors glucose deprivation and lactic acidosis can induce tumor CIN and propose that they are potentially responsible for human tumor aneuploidy.

## Introduction

Chromosomal instability (CIN) is now recognized as a driving force for cancer initiation and progression [1]. Aneuploidy often exists in precancerous lesions [2], [3] and carcinoma in situ [4]. CIN is a prominent feature of human tumors [5]. There is a correlation of chromosomal aberration with tumor grade and prognosis [6], [7].

Mechanistically, CIN is caused by abnormal mitosis, typified by abnormalities in dynamics of microtubule/centrosome, mitotic timing (early exit or lagging), and mitotic checkpoint control, among others. Any errors in these events may ultimately result in the inability of cancer cells to faithfully segregate sister chromatid to daughter cells. These events are influenced by either the intrinsic or the extrinsic factors. The genes (intrinsic factors) that play key roles in these events have been studied in depth. However, the extrinsic factors, such as those in tumor microenvironment, which may significantly influence mitosis of cancer cells, have not been extensively and intensively studied yet.

Cancer cells in solid tumors are surrounded by a hostile environment featured with nutrient shortage, lactic acidosis, hypoxia, etc, such that tumor cells are temporally or constantly under stress [8], [9], [10], [11], [12], [13]. There are reports regarding the effect of hypoxia, acidosis, and glucose deprivation on genetic instability such as gene mutation [14]. In addition, Morita T., et al., demonstrated that acidosis, particularly at pH lower than 6.5, could induce sister-chromatid exchanges and chromosomal aberrations in various cultured mammalian cells, and the effect was S-phase dependent [15], [16].

On the basis of the rationales and facts described above, we investigated the potential effect of glucose deprivation and lactic acidosis, two prominent tumor environmental factors, on cancer CIN.

## Materials and Methods

### Cell culture

Murine breast cancer cell line 4T1(*p53-null*), human breast cancer cell line MCF-7(*p53+/+*) and human colon carcinoma cell line HCT116 (*p53+/+*) were maintained in complete RPMI-1640 (Life Technologies, USA) with 10% FBS, 100 U/ml penicillin/streptomycin and 2 mM L-glutamine. All cell lines were obtained from and characterized by The Cell Bank of Type Culture Collection of Chinese Academy of Sciences according to the cell line authentification testing (vitality, species confirmation and interspecies contamination, DNA fingerprinting and mycoplasma contamination ), and were used within 6 months after resuscitation.

### Stress condition

In order to create metabolic stress condition which would sustain survival of cancer cells, we cultured 1×10^6^ 4T1 or MCF-7 cells in medium containing 3 mM glucose (HCT116 was cultured in medium containing 0.5 mM glucose) with lactic acidosis for 7 days. These cells were under severe metabolic stress but still alive. Then, the surviving cells were cultured in fresh medium containing 3 mM glucose with lactic acidosis for another 48 hours to allow sufficient growth recovery. Lactic acidosis was generated by adding pure lactic acid (Sigma-Aldrich, Switzerland) to the culture media to a final lactate concentration at 20 mM and pH at 6.7, as previously described [17]. The pH values and lactate concentrations used here are in physiological ranges in solid tumors, as intratumoral pH can be as low as 6.0 and intratumoral lactate can vary between 3–40 mM [18], [19], [20], [21], [22], [23], [24], [25].

### Cell count, glucose and lactate measurement

Cell count was carried out with a hematocytometer under an optical microscope. Glucose in the culture medium was quantified by hexokinase colorimetric method using Olympus AU2700 system (Olympus, Tokyo, Japan). Lactate in culture medium was determined by VITROS Chemistry Product LAC Slides using VITROS 5.1 FS system (Ortho Clinical Diagnostics, Raritan, NJ).

### The cytokinesis-block micronucleus (CBMN) assay

The CBMN assay is one of the most commonly used methods for measuring chromosome instability [26]. The experimental procedure is based on the method as described previously by French [27]. Cells were cultured in medium containing 4.5 µg/ml cytochalasin B (Sigma, USA) to block cytokinesis for 24 hours, trypsinzed, spun onto slides using a cytospin cytocentrifuge (Shandon Scientific, UK), air-dried, fixed in 3∶1 methanol/glacial acetic acid and stained with Wright and Giemsa. Binucleated (BN) cells, micronuclei (MNi), nucleoplasmic bridges (NPBs) and nuclear blebs (NBs) were scored as described by Camps et al [28]. MNi were morphologically identical, their diameters varies between 1/16 and 1/3 of the mean diameter of the main nuclei, not linked to the main nuclei. NPBs were continuous nucleoplasmic bridges between the two nuclei in a BN cell, which is no wider than 1/4 of the nuclear diameter. NBs were small protrusions of the nuclear material, connected to the main nucleus by a thin chromatin segment. For each sample, a total of 1000 BN cells were evaluated for the frequency of MNi, NPBs and NBs.

A total of 500 living interphase cells were used for assessment of mono-, bi-, and multi- nucleated cells without adding cytochalasin B.

### Fluorescence In Situ Hybridization (FISH)

FISH was performed using pan-centromeric probes which recognize all chromosomes (KREATECH Diagnostics, The Netherlands). Chromosome-specific centromeric probes (FITC -conjugated alpha-satellite DNA probes for chromosomes 7 and TRITC -conjugated probes for chromosomes 17) were from Cytocell (UK). Molecular hybridization and immunofluorescent detection were carried out according to the manufacturer's instructions. Fluorescence was observed under Zeiss LSM 710 laser confocal microscope equipped with Zen software to process the image.

### Immunofluorescent Confocal Laser Microscopy

Cells on coverslip were fixed with 4% paraformaldehyde in PBS, permeabilized with 0.1% Triton X-100 in PBS, and blocked with 2% bovine serum albumin in sodium phosphate buffer (pH 7.4). Cells were co-stained with anti-pericentrin (1∶500 dilution of ab4448, Abcam, UK) and anti-alpha tubulin (1∶500 dilution, ab7291, Abcam, UK) or anti-γ-H2AX(1∶100 dilution, ab22551, Abcam, UK) and anti-53BP1(1∶100 dilution, 4937s, Cell signalling, USA) according to the protocols of the suppliers. Alexa Fluor 633-conjugated goat anti-mouse and Alexa Fluor 488-conjugated goat anti-rabbit antibody (1∶500) were from Molecular Probes (Invitrogen, USA). Nuclei were counterstained with DAPI. Fluorescence was observed under Zeiss LSM 710 laser confocal microscope equipped with Zen software to process the image.

### Cell cycle assay and immunofluorescent detection of phosphorylated histone H3

1×10^6^ cells were collected and fixed in 70% ethanol (4°C, 48 hours). After fixation, the cells were washed with PBS, and permeabilized with 0.3% Triton X-100 in PBS for 15 min. Then the cells were blocked in 1% BSA for 10 min and stained with antibody that specifically recognizes the phosphorylated form of histone H3 (1∶1000 dilution, 3377s, Cell signalling, USA). After incubated for 1 hr in room temperature, the cells were rinsed with PBS and incubated with FITC-conjugated IgG antibody for 30 min. Then the cells were rinsed with PBS twice, and stained with the CycleTEST™ PLUS DNA Reagent Kit (BD Biosciences, USA). The samples were analyzed by the FACScan™ Calibur (Becton Dickinson, USA). The FCS Express version 3 (De Novo Software) was utilized to analyze the data.

### Quantitative real-time reverse transcription–PCR

Total RNA was extracted using RNeasy Mini Kits (Qiagen, USA). The mRNA was reverse transcribed into cDNA using the M-MLV Reverse Transcriptase (Promega, USA). Then, 20 ng of cDNA was subjected to quantitative real-time PCR analyses targeting Mad2, Bub1b, Bub1, Bub3, Cdc2 and Cyclin B1 using the SYBR® Premix Ex Taq™ (TaKaRa, China). The primer sequences were listed in Table S1. Analysis was performed using the StepOne Real-Time PCR System and the StepOne v2.0 software (Applied Biosystems, Germany). Data was presented as the fold difference in the investigated genes expression normalized to gene GAPDH as endogenous reference, relative to the untreated control cells.

### Western blot analysis

Cells were lysed with M-PER mammalian protein extraction reagent (Pierce, USA), supplemented with protease inhibitor cocktail (Pierce, USA). Protein concentration was measured by BCA protein assay (Pierce, USA). After heat denaturation, samples were stored at −80°C before use. The protein was applied to a 10% to 12% SDS polyacrylamide gel, transferred to a PVDF membrane, and then detected by the proper primary and secondary antibodies before visualization by Western Lighting Plus ECL kit (Perkin Elmer, USA). The primary antibodies used: rabbit anti-mad2, rabbit anti-bub1b, rabbit anti-bub3 and rabbit anti-cdc2 (Cell Signalling Technology, USA), rabbit anti-cyclin B1 and mouse anti-bub1 (Sigma, USA).

### Statistical analysis

Unless otherwise stated, experiments were done three times; data were expressed as mean ± SD. Comparisons between groups were evaluated using two tailed Student t tests.

## Results

### A glucose deprivation model

**Figure 1:**
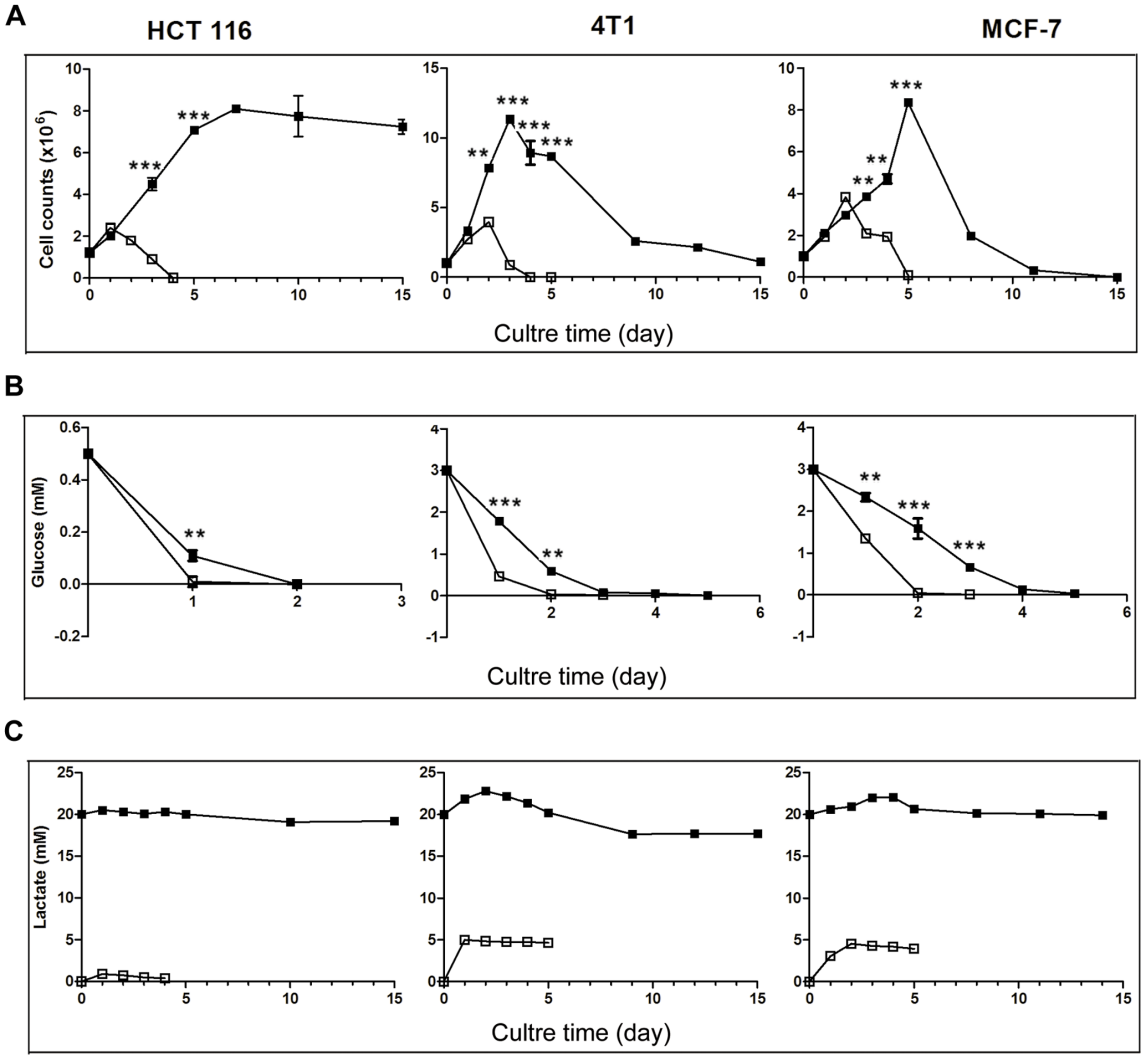
Lactic acidosis rescues HCT116, 4T1 and MCF-7 cells from glucose deprivation. 4T1 or MCF-7 cells were cultured in RPMI-1640 containing 3 mM glucose with or without lactic acidosis. HCT116 cells were cultured in RPMI-1640 containing 0.5 mM glucose with or without lactic acidosis. Cell count, lactate and glucose in culture medium were determined as described in Materials and Methods. (**A**) (**B**) & (**C**) Curves of cell growth/death, glucose consumption, and lactate generation. Solid symbol, with lactic acidosis; open symbol, without lactic acidosis. * p<0.05, **, p<0.01, *** p<0.005, as compared with control.

Severe metabolic stress such as glucose deprivation rapidly kills cancer cells. To gain CIN, cancer cells must survive through metabolic stress. We recently found that cancer cells under lactic acidosis can tolerate glucose deprivation. We added pure lactic acid to the culture media to final concentration of 20 mM with a corresponding pH of 6.7, as previously described [17]. The pH values and lactate concentrations used were in the physiological ranges in solid tumors [18], [19], [20], [21], [23], [25]. 4T1 and MCF-7 cells were cultured in RPMI-1640 containing 3 mM glucose with or without lactic acidosis. HCT116 cells were cultured in RPMI-1640 containing 0.5 mM glucose with or without lactic acidosis. Without lactic acidosis, cells died rapidly when glucose was exhausted. With lactic acidosis, cells' survival time was significantly extended after glucose was used up (Figure 1). The results showed that lactic acidosis conferred cancer cells with ability to survive under glucose deprivation, consistent with our previous report [17].

### Glucose deprivation and lactic acidosis induce CIN

**Figure 2:**
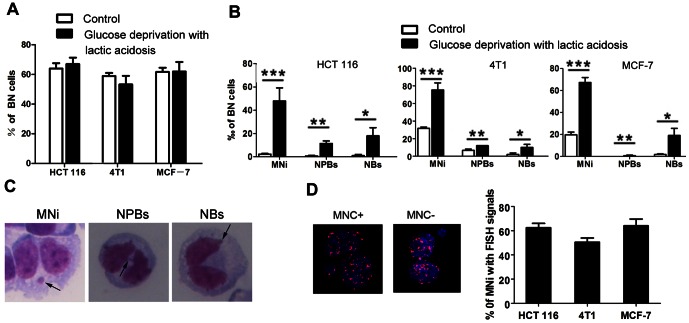
One cycle of glucose deprivation with lactic acidosis followed by nutrient restoration significantly increases micronuclei (MNi), nucleoplasmic bridges (NPBs), and nuclear blebs (NBs) in HCT116, 4T1 and MCF-7 cancer cells. (**A**) Percentage of binucleated (BN) cells in the cells that were cultured for 7 days as described in Figure 1 and resume mitosis upon nutrient restoration in the presence of cytochalasin B. Control cells are maintained in regular medium without lactic acidosis. (B) The numbers of cells that carry MNi, or NPBs, or NBs in every 1000 BN cells. *p<0.05, **p<0.01, *** p<0.005, in comparison to control. MNi, NPBs and NBs were determined by cytokinesis block micronucleus (CBMN) assay as described in Materials and Methods. (**C**) Representative photos of MNi, NPBs, and NBs. (**D**) MNi that contains chromosomes by pancentromeric FISH probes. MNC+, MNi containing whole chromosome; MNC−, MNi containing chromosomal accentric fragment.

The cells cultured in medium containing 3 mM glucose with lactic acidosis for 7 days (Figure 1) were under glucose deprivation, as glucose was already exhausted on day 2 (HCT116), on day 3 (4T1), or on day 5 (MCF-7). Upon nutrient restoration, these cells resumed mitosis, as manifested by the binuclear cells, which were 62.5±3.5% (HCT116), 53.33±5.77% (4T1), and 62.0±6.45% (MCF-7) (Figure 2A), comparable with those maintained in regular culture medium. We then checked if these cells surviving through glucose deprivation had increased CIN during the mitotic recovery. The results showed that the frequencies of MNi, NPBs, and NBs, the indicators of CIN, were significantly increased (Figure 2B&C). Pancentromeric FISH probes were used to detect the percentage of micronuclei that carry whole chromosomes and chromosomal accentric fragment (Figure 2D). The results indicated that there existed both whole-chromosome loss and DNA breakage in cells.

**Figure 3:**
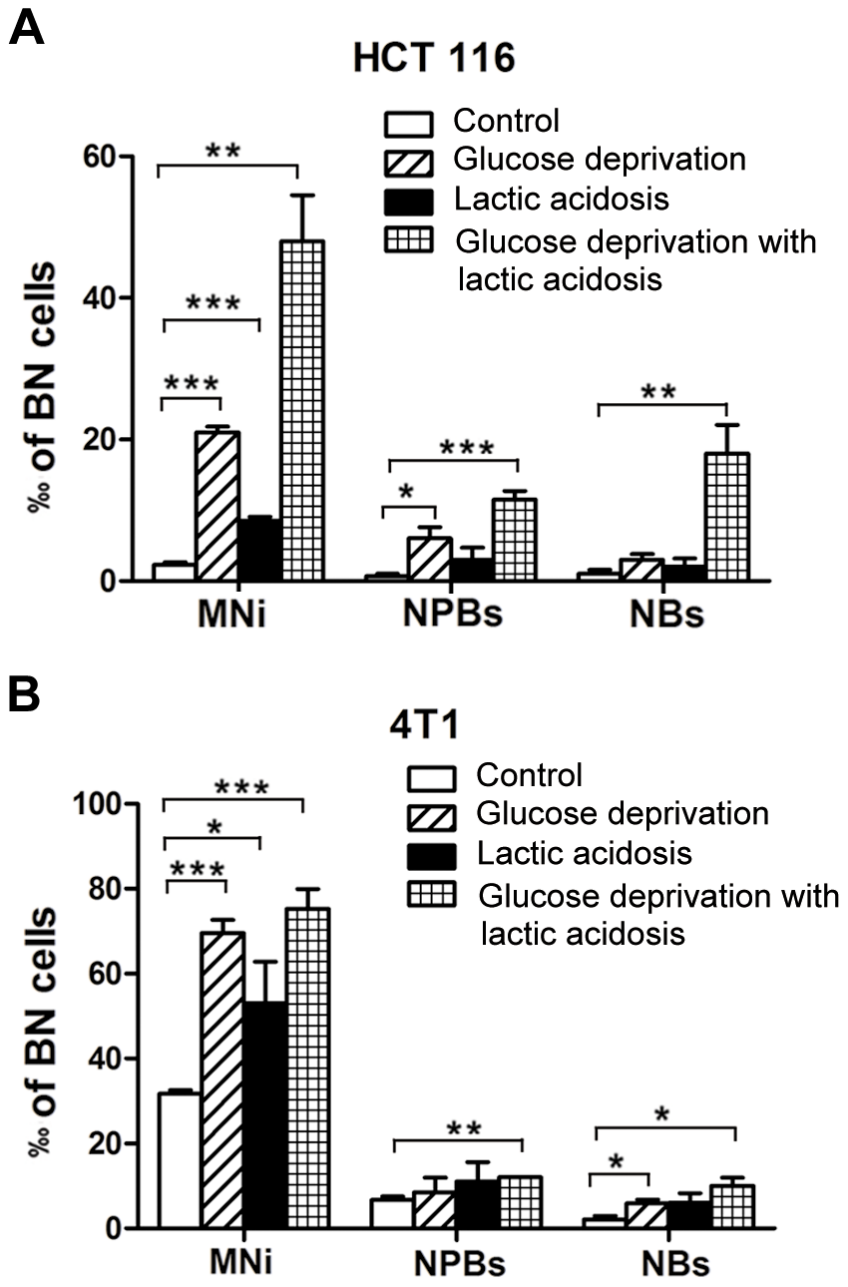
The effect of lactic acidosis, glucose deprivation, and lactic acidosis with glucose deprivation on chromosome instability. (A) The numbers of HCT116 cells that carry MNi, or NPBs, or NBs in every 1000 BN cells. *p<0.05, **p<0.01, *** p<0.005. HCT116 cells were cultured in 4 different conditions: condition 1 (control), regular RPMI-1640; condition 2 (glucose deprivation), RPMI-1640 containing 0.5 mM glucose; condition 3 (lactic acidosis), regular RPMI-1640 with 20 mM lactic acidosis. To avoid glucose deprivation during 7-day culture, we replaced the medium every 2 days; condition 4 (glucose deprivation with lactic acidosis), RPMI-1640 containing 0.5 mM glucose with 20 mM lactic acidosis. Cells were harvested at the indicated time and subjected for analysis of MNi, or NPBs, or NBs. (**B**) The numbers of 4T1 cells that carry MNi, or NPBs, or NBs in every 1000 BN cells. *p<0.05, **p<0.01, *** p<0.005, in comparison to control. 4T1 cells were cultured in 4 different condition as described in (A) except condition 2 and 4 in which glucose was 3 mM.

Lactic acidosis and glucose deprivation represents 2 separate stresses for cancer cells. We then determined the MNi, NPBs, and NBs of HCT116 and 4T1 cells exposed to glucose deprivation or lactic acidosis. For glucose deprivation, HCT116 cells were incubated in culture medium containing 0.5 mM glucose for 3 days then collected for determination of MNi, NPBs, and NBs, because cells on day 3 were deprived of glucose, given the fact that glucose was exhausted on day 1 and cells died out on day 4 (Fig. 1A & B). For lactic acidosis exposure, cells were cultured in regular culture medium containing 6 mM glucose supplemented with 20 mM lactic acid. In order to avoid glucose deprivation, we replaced the medium every 2 days. The results demonstrated that both glucose deprivation and lactic acidosis increased CIN, but combination of glucose deprivation with lactic acidosis apparently achieved an additive effect (Figure 3A). Glucose deprivation or lactic acidosis also caused an increase of CIN in 4T1 cells, but the effect of combination of glucose deprivation with lactic acidosis on CIN was not further enhanced (Figure 3B). The results thus indicated that both lactic acidosis and glucose deprivation could induce CIN, but the additive effect of glucose deprivation with lactic acidosis depends on cell lines. This difference may be associated with genetic background, e.g., HCT116 has intact p53 and near diploid, whereas 4T1 is p53 null and show a complex aneuploid karyotype, and the basal levels of MNi, NPBs, and NBs are significantly higher in 4T1 than in HCT116 (Figure 3A&B).

**Figure 4:**
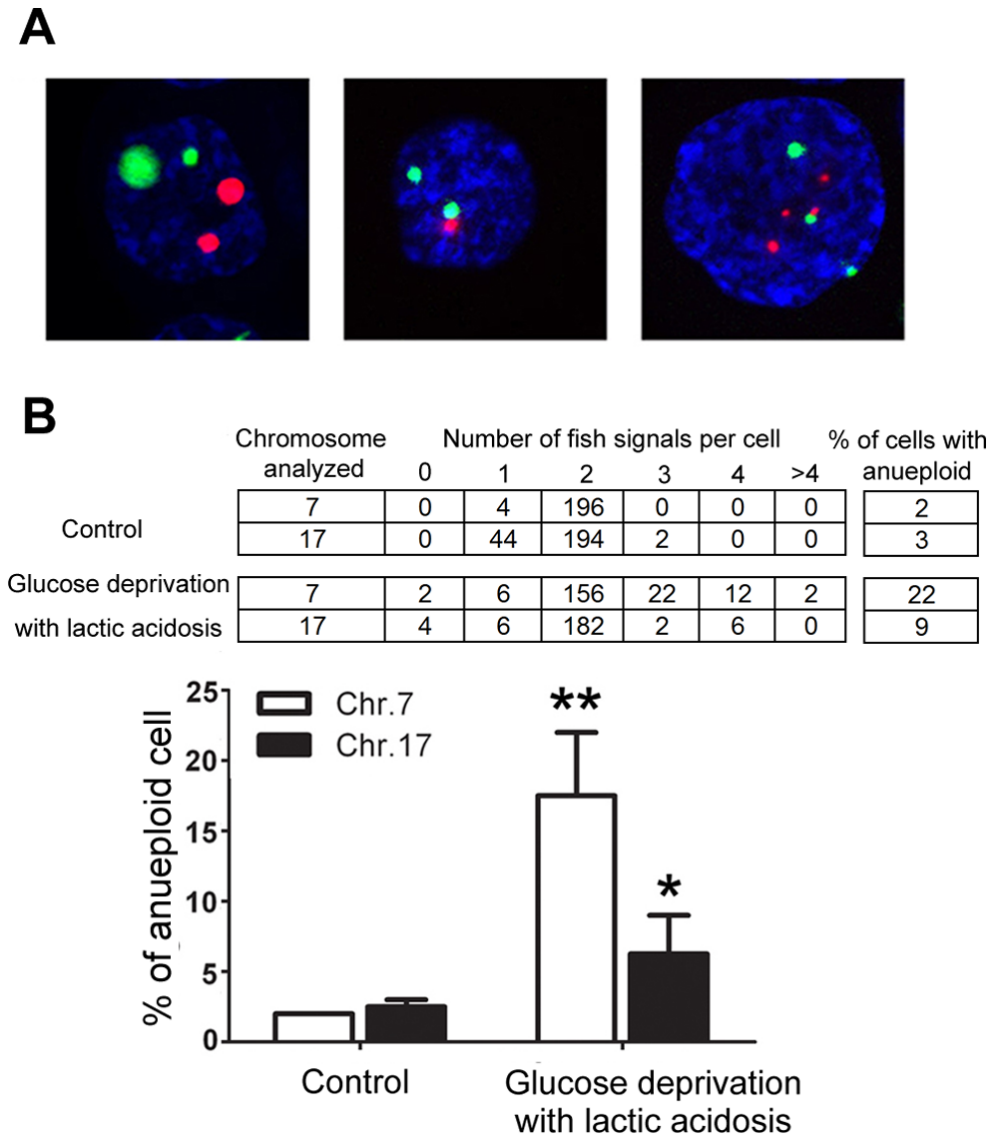
One cycle of glucose deprivation with lactic acidosis followed by nutrient restoration significantly increases aneuploidy in HCT116 cells. Cells were cultured in RPMI-1640 medium containing 0.5 mM glucose with lactic acidosis for 7 days. The cells surviving through glucose deprivation were then cultured in fresh medium for 48 hours and subjected for aneuploid analysis. Control cells were maintained in regular RPMI-1640 medium without lactic acidosis. (**A**) Representative photos of diploid and aneuploid cells scored by chromosomes 7(green) and 17(red). (**B**) Percentage of aneuploid cells scored by chromosome 7 or 17. *p<0.05, **p<0.01, as compared with control. The results were confirmed by 2 independent experiments.

Because MCF-7 and 4T1 cell lines have a complex aneuploid karyotype, they are not suitable models for scoring aneuploid generation. HCT116 is a human colon cancer cell line with a stable near-diploid karyotype hence we used it to evaluate the effect of glucose deprivation with lactic acidosis on aneuploid generation. HCT116 cells were cultured in medium containing 0.5 mM glucose with lactic acidosis for 7 days, then cultured in fresh RPMI-1640 medium for 48 hours and scored for anueploidy by FISH using chromosome 7- and 17-specific centromeric probes. The aneuploid cells as judged by chromosome 7 and 17 were increased by 11 and 3 folds, respectively, in comparison to control (Figure 4). Notably, polysomy of chromosomal 7 is frequently observed in many types of human tumors [29], [30].

Other lines of evidence that demonstrated the effect of glucose deprivation with lactic acidosis on cancer cell CIN included misaligned chromosome in the metaphase, lagging chromosome in the anaphase, and nucleoplasmic bridge in the telophase (Figure S1).

### Glucose deprivation with lactic acidosis induces centrosome amplification, multipolar mitosis, and multinucleation

**Figure 5:**
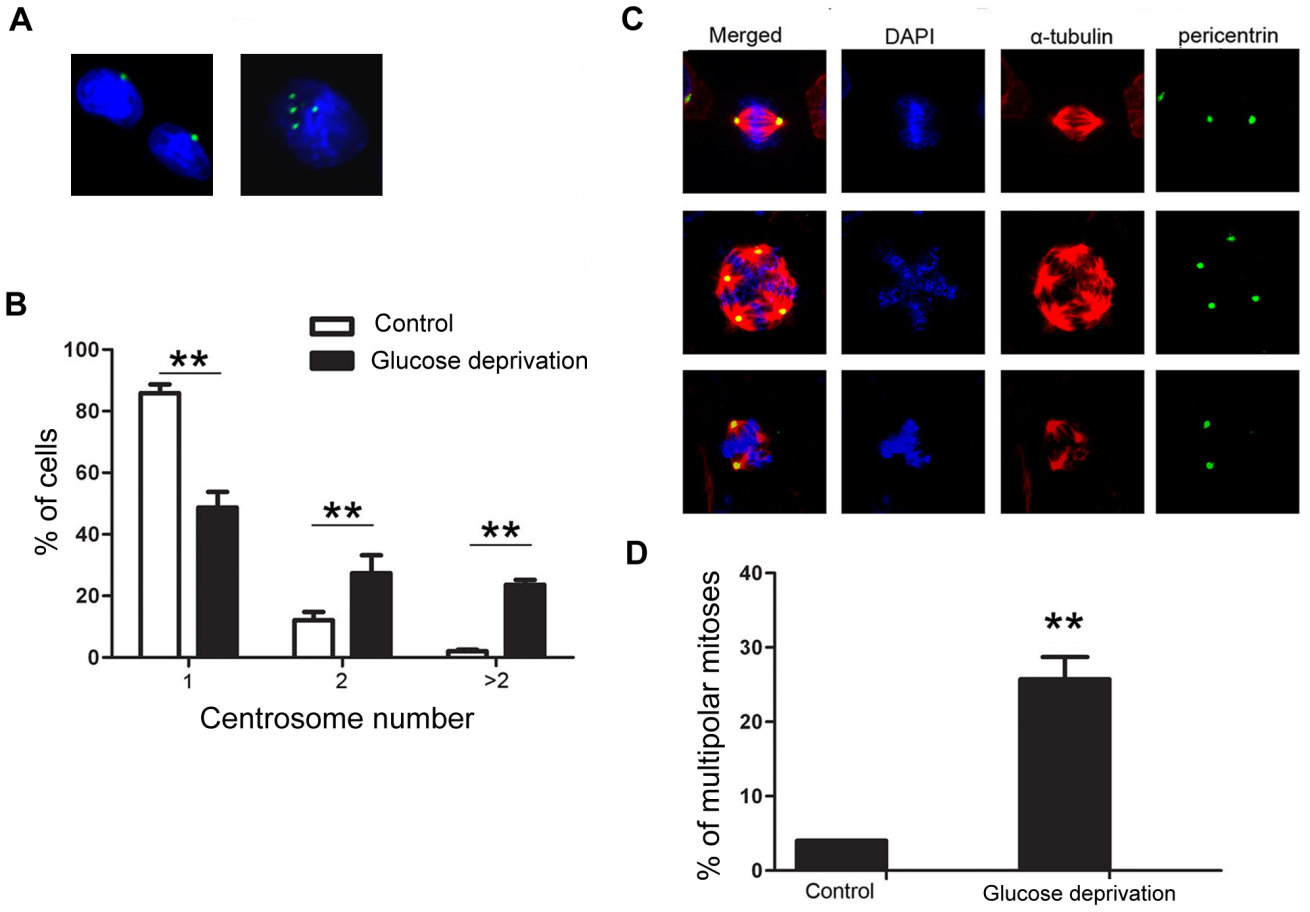
One cycle of glucose deprivation with lactic acidosis followed by nutrient restoration induces centrosome amplification and multipolar division in 4T1 cells. Cells were cultured in RPMI-1640 medium containing 3 mM glucose with lactic acidosis for 7 days. The cells surviving through glucose deprivation with lactic acidosis were then cultured in fresh medium for 48 hours and subjected for analysis of centrosome and spindle. Control cells were maintained in regular RPMI-1640 medium without lactic acidosis. (A) Representative photos of nonmitotic cells with one or multiple centrosomes. (B) The percentage of cells with multiple centrosomes (>1) in the population of nonmitotic cells (pericentrin, green; DAPI, blue). **p<0.01, in comparison to control. (C) Representative photos of mitotic cells with bipolar or multipolar spindles. (D) The percentage of cells with multipolar spindles in the population of mitotic cells. **p<0.01, in comparison to control.

**Figure 6:**
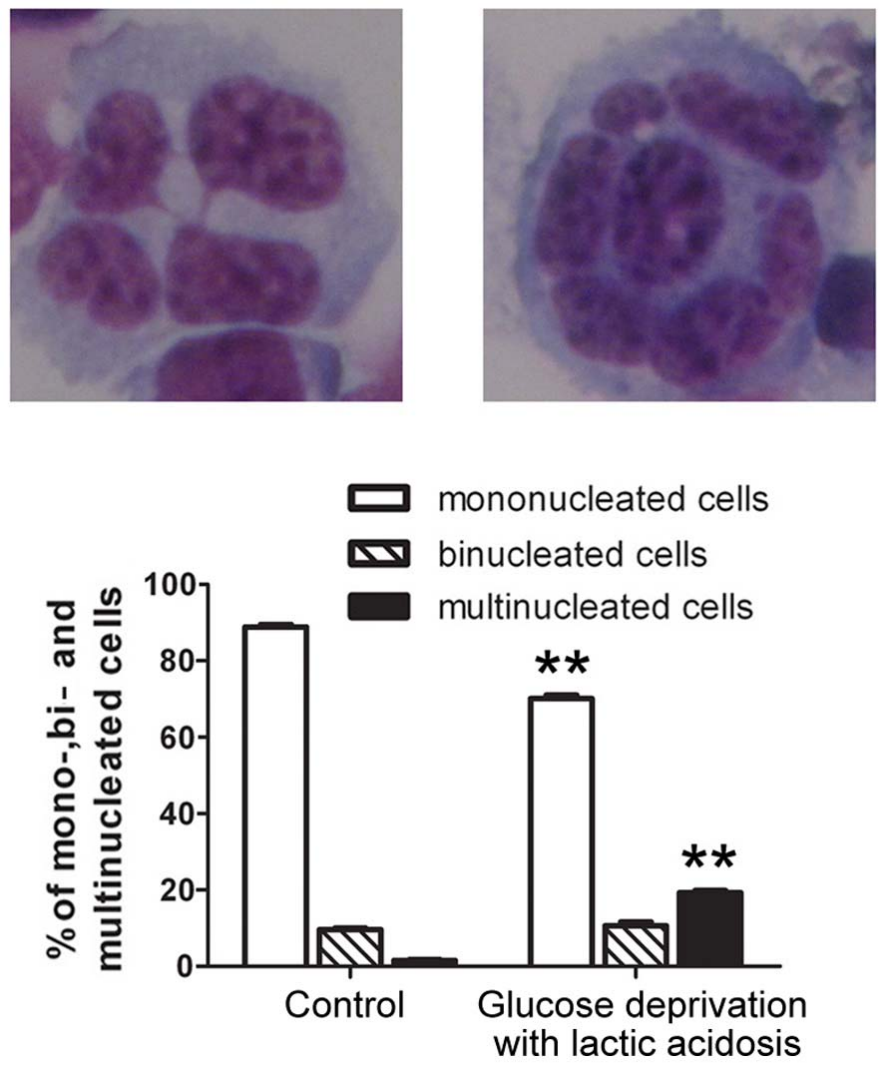
One cycle of glucose deprivation with lactic acidosis followed by nutrient restoration significantly increases multinucleation in 4T1 cells. **p<0.01, in comparison to control. Cells were cultured in RPMI-1640 medium containing 3 mM glucose with lactic acidosis for 7 days. The cells surviving through glucose deprivation with lactic acidosis were then cultured in fresh medium for 48 hours. Control cells were maintained in regular RPMI-1640 medium without lactic acidosis.

Since centrosome amplification is strongly associated with chromosomal instability/spindle multipolarity/multinucleation in human tumor [31], [32], [33], we examined if glucose deprivation with lactic acidosis could induce centrosome amplification in cells. Our results showed that the treatment exerted a significant effect on centrosome amplification, as manifested by a more than 10-fold and 6-fold increase of cells with multiple centrosomes at both non-mitotic (Figure 5A & B) and mitotic phase (Figure 5C & D), respectively. The amplification of centrosome was accompanied with multipolar mitosis (Figure 5C & D). Although multipolar spindles generally lead to mitotic cell death, they could form a functional pseudo-bipolar spindle if clustered to opposite poles [34]. Such functional spindle is associated with melotelic attachment (a single kinetochore is attached to both opposite spindle poles), which leads to lagging chromosome and segregation errors [34]. Meanwhile, the multinucleated cells increased by 12-fold as compared with control (Figure 6), a consequence related to multipolar mitosis or successive cycles of bipolar mitosis without cytokinesis.

However, centrosome amplification, multipolar mitosis, and multinucleation were not observed in MCF-7 and HCT116 cells. This difference might be caused by *p53*. 4T1 is *p53* null, whereas MCF-7 and HCT116 are *p53* wild-type. Previous reports showed that multiple centrosomes, multipolar mitosis, and cytokinesis failure could arise from failure of *p53*-dependent tetraploidy checkpoint [35].

### Glucose deprivation with lactic acidosis induces a dysregulation of mitotic checkpoint

**Figure 7:**
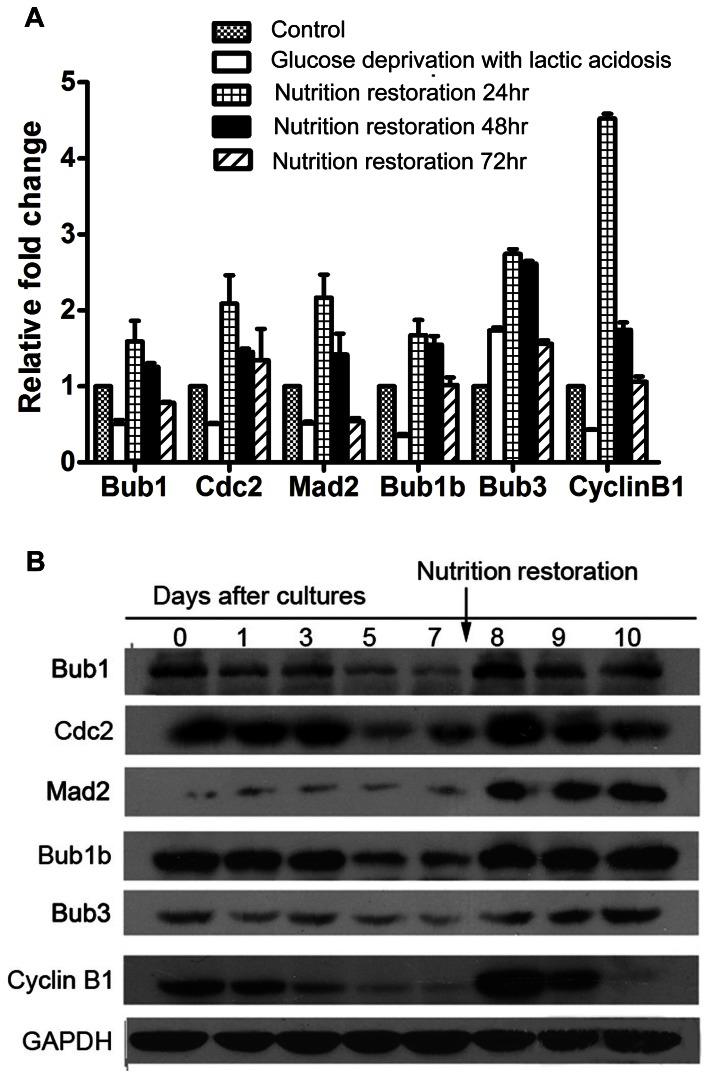
The effect of glucose deprivation with lactic acidosis followed by nutrient restoration on key members of mitotic checkpoint in HCT116 cells. Cells were cultured in RPMI-1640 medium containing 0.5 mM glucose with lactic acidosis for 7 days. The cells surviving through glucose deprivation with lactic acidosis were then cultured in fresh medium. Cells were collected at indicated time for real-time PCR (A) and Western Blot (B) analysis. The results were confirmed by 2 independent experiments.

Since hyperactivated mitotic checkpoint is another major mechanism that leads to human tumor CIN [1], [36], [37], [38], [39], [40], we tested if glucose deprivation with lactic acidosis could disturb the expression of the mitotic checkpoint genes such as mad2, bub1, bub3, and bub1b. According to the PCR result (Figure 7A), the mRNA levels of these components except Bub3 significantly decreased when glucose was deprived. Upon nutrition restoration, all the components increased to a level significantly exceeding the initial levels, then declined. The results indicated that, at the transcription levels, these components of the mitotic checkpoints, were disturbed. We then checked the protein levels of these components (Figure 7B). During glucose deprivation, except mad2, the expression of bub1, bub3, bub1b, cdc2 and cyclin B1 significantly decreased (Figure 6A). Upon nutrient restoration, expression of all 6 proteins was increasing. Notably, the amount of mad2 was 6-fold (analyzed by the densitometry of the Western blot) higher than that of control cells – the cells maintained in regular culture, suggesting that mitotic recovery of cells surviving through glucose deprivation was accompanied with an abnormal high expression of Mad2. Nevertheless, the levels of the mRNA and proteins apparently did not match very well with each other, e.g., mRNA levels of Mad2, Bub1b, and Bub3 declined on day 2 after nutrient restoration, whereas protein levels of these components did not, suggesting a potential post-transcriptional regulation of these components. Taken together, the results suggest that glucose deprivation with lactic acidosis induces cancer cell CIN potentially via its disturbing mitotic checkpoint, although validating the relationship needs further studies.

**Figure 8:**
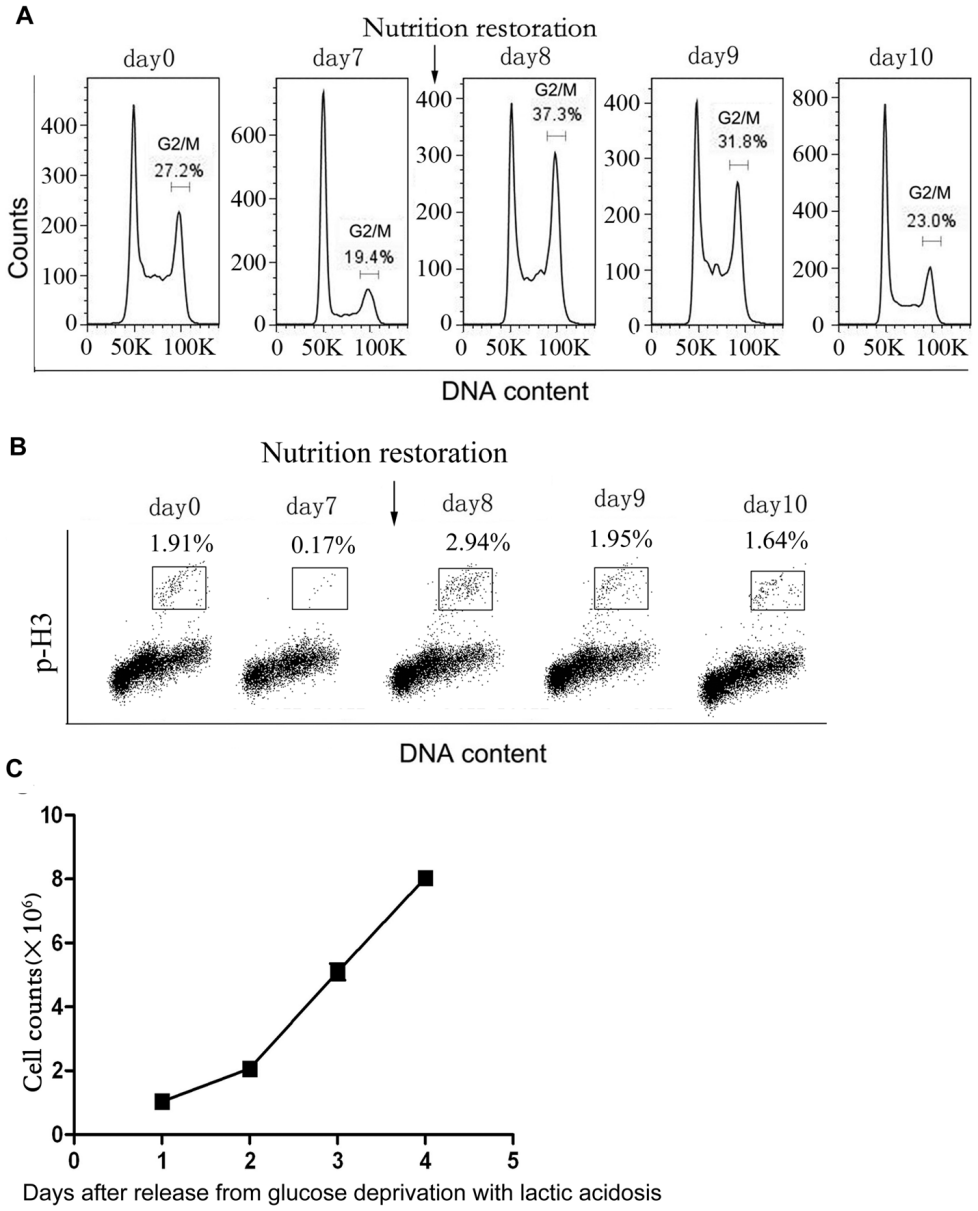
The effect of glucose deprivation with lactic acidosis followed by nutrient restoration on cell cycle in HCT116 cells. Cells were cultured in RPMI-1640 medium containing 0.5 mM glucose with lactic acidosis for 7 days. The cells surviving through glucose deprivation with lactic acidosis were then cultured in fresh medium. (A) & (B) Cells were collected at indicated time for analysis of cell cycle and phospho-Histone H3 labelling analysis. (C) The cell growth after release from stress upon nutrition restoration. The results were confirmed by 2 independent experiments.

Since it was previously shown that overexpression of Mad2 led to prolongation of M phase [37], we checked the percentage of G2/M population in the cells that recovered mitosis after stress. G2/M percentage in these cells was significantly higher than that of control cells (cells under regular culture) (Figure 8A), followed by a decrease to the initial level (day 0). Consistently, the percentage of cells with mitotic marker (phosphor-Histone H3) [41] were increased on the first day upon nutrient restoration followed by a decline back to the initial level (day 0) (Figure 8B). Consistently again, the growth slope of the cells on the first day of nutrient restoration was shallow as compared with the next 2 days (Figure 8C).

The expression profile of Mad2, Bub1b, Bub1, and Bub3 at mRNA levels matches well with the data of cell cycle (Figure 7A, Figure 8), suggesting a coordination of cell cycle with the mitotic checkpoints in cells that underwent the stress and release. Nevertheless, protein levels of Mad2, Bub1B, and Bub3 remained high (Figure 7B) even after stress release (day 2 and day 3 after nutrient restoration). We suggest that there is a lagging time for these proteins. The puzzling question is: if the high levels of these components were responsible for the temporary arrest at G2/M in the first day after stress release, how they could permit G2/M release on day 2 and 3 after nutrient restoration. Would it be possible on day 2 and 3 after nutrient restoration, these proteins did not form functional quaternary structure? This is an issue we are considering.

### Glucose deprivation with lactic acidosis increases DNA damage

**Figure 9:**
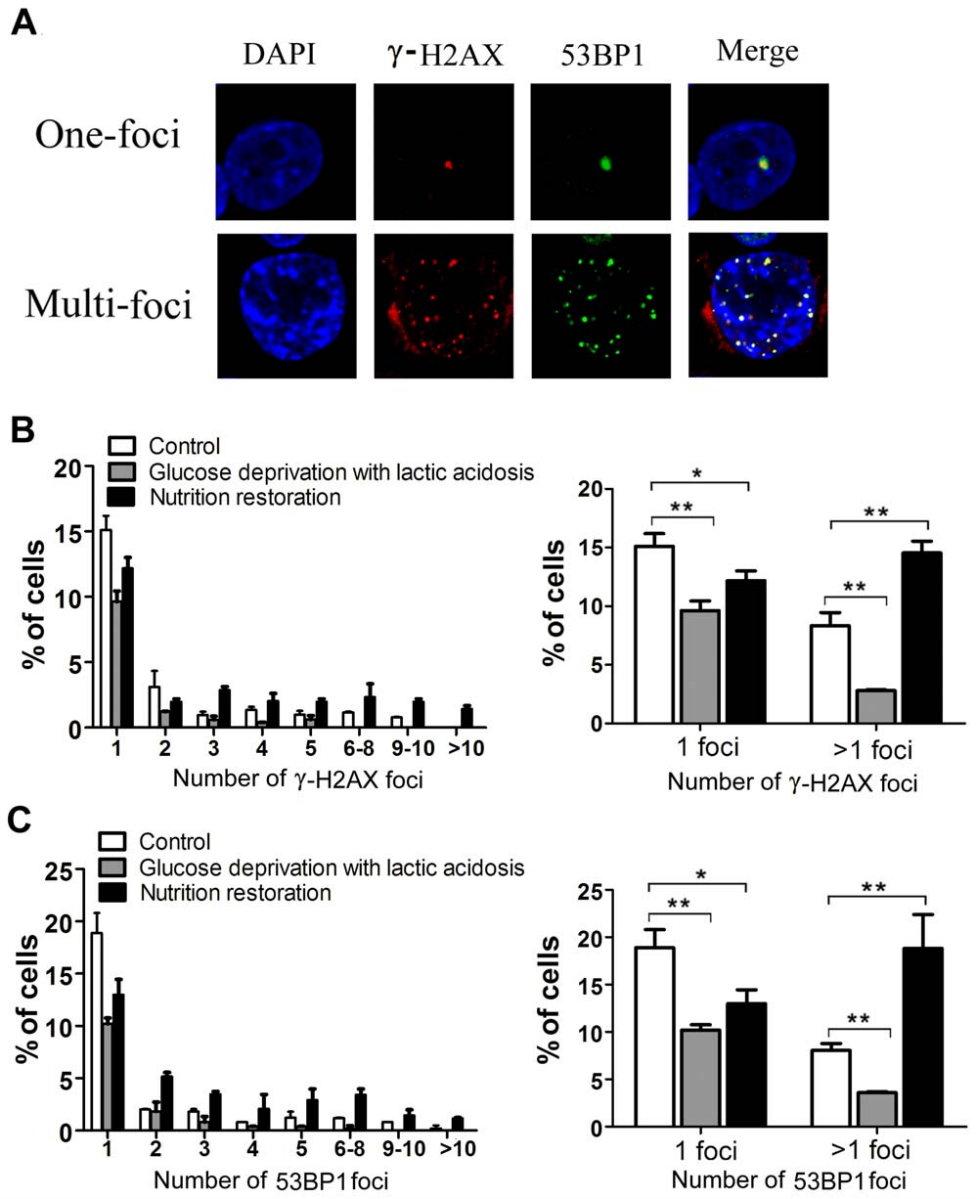
The effect of glucose deprivation with lactic acidosis followed by nutrient restoration on DNA damage in HCT116 cells. Cells were divided into 3 groups. Group 1 (control), cells were cultured in regular RPMI-1640 medium; Group 2 (glucose deprivation with lactic acidosis), cells were cultured in RPMI-1640 medium containing 0.5 mM glucose with lactic acidosis for 7 days; Group 3 (nutrition restoration), cells were cultured in RPMI-1640 medium containing 0.5 mM glucose with lactic acidosis for 7 days then cultured in fresh medium for 48 hours. DNA damage was scored by γ-H2AX and 53BP1 labelling as described in Materials and Methods. (A) Representative photos of cells with γ-H2AX and 53BP1 labelling. (B) Number of γ-H2AX per cell (n = 300) and statistical analysis. (C) Number of 53BP1 foci per cell (n = 300) and statistical analysis. *p<0.05, **p<0.01, in comparison to control.

Another possible explanation for the increased MN, NPBs, and NBs, and potentially also for supernumerary centrosomes and aneuploidy generation, is an increase in DNA damage as a result of glucose deprivation with lactic acidosis. We checked the levels of DNA damage in cells exposed to glucose deprivation with lactic acidosis followed by nutrient restoration, using immunofluorescence staining for γ-H2AX and 53BP1 [42], [43], scored according to Lukas et al [44]. After DNA double strand break (DSB), histone H2AX surrounding DSB would be phosphorylated. The phosphorylated H2AX was termed γ-H2AX. DSB also recruit the DNA damage sensor p53-binding protein 1 (53BP1), which was retained by γ-H2AX. Thus, the staining of γ-H2AX and 53BP1 was mostly overlapping (Figure 9A). The results indicated that under glucose deprivation with lactic acidosis, DNA damage was even lower than control. This was not surprising, as these cells were at G0/G1 phase [17], in which DNA synthesis was inactive. However, after release from stress upon nutrient restoration, DNA damage in cells, as reflected by foci per cells greater than 1, increased significantly (Figure 9B).

## Discussion

There are in general 3 mechanisms underlying aneuploid human tumours as summarized by Schvartzman et al [1]: (a) Loss/mutation/downregulation of mitotic checkpoint is considered to be associated with tumor CIN, as the weakened checkpoint may allow premature exit from mitosis and premature separation of sister chromatids. Although many studies using mouse models demonstrated that mutation/loss/downregulation of mitotic checkpoint members indeed could lead to tumor CIN [45], it is increasingly recognized that mutation/loss/downregulation of mitotic checkpoint members is rare based on extensive analysis of aneuploid human tumours. (b) In most cases, these genes in aneuploid human tumours are upregulated [46]. It has been shown that overexpression of these genes is sufficient to generate aneuploidy and to initiate tumourigenesis [47]. Mechanistically, hyperactive mitotic checkpoint tends to prolong mitosis and to increase the chance of merotelic attachment and lagging chromosome. (c) Centrosome amplification appears to be an alternative mechanism responsible for human aneuploid tumours [1]. Multiple centrosomes in cancer cells can cluster at 2 poles to form a pseudo-bipolar spindle. Such spindle, however, increases frequency of lagging chromosome and segregation errors in mitosis [48], [49]. Therefore, hyperactivated mitotic checkpoints and centrosome amplification are increasingly recognized as the major mechanistic basis responsible for aberrant mitosis and CIN in human tumours.

How mitotic checkpoint in human tumours is upregulated is not completely known. We show that glucose deprivation with lactic acidosis can upregulate the expression of mitotic checkpoint genes. Interestingly, human tumor CIN shares similar feature as that induced by glucose deprivation with lactic acidosis, such as aneuploidy, spindle multipolarity, multinucleation, hyperactivated mitotic checkpoint and/or abnormal amplification of centrosome. The similarity suggests that glucose deprivation and lactic acidosis are environmental factors relevant to aneuploid human tumours. Besides, glucose deprivation and lactic acidosis can also cause DNA damage, which is another potential way leading to cancer CIN. Solid tumor, particularly poorly-vascularized tumors, is spatially or temporally under stress [10], [12], [50], [51]; lactic acidosis, a tumor environmental factor, can transit cancer cells to a ‘dormant’ like state under glucose deprivation [17]; when glucose/nutrient is provided, these cells could resume mitosis but with a higher frequency to acquire CIN.

We did not further study the fate of the cells that acquire CIN imposed to glucose deprivation with lactic acidosis, because previous literatures have clearly shown the destinies for those cells, whose survival or death follows ‘the fittest survival’ principle. While some die under the sustained stress environment [52], [53], others may acquire growth advantage, for example, overexpression of EGFR due to polysomy of chromosome 7 may maintain basal glucose uptake and support cancer cells survival in the low glucose medium [54]. We noted that the numbers of chromosome 7 in the aneuploid HCT116 cells induced by glucose deprivation with lactic acidosis followed by nutriontion restoration ranged from 0–6, suggesting the different environmental adaptability of these cells.

Targeting hyperactivated mitotic checkpoint is an approach to treat tumor. Candidate drugs targeting CENPE, CDC20, Aurora kinase, etc, are under clinical trials [55], [56]. Another potential alternative approach could be manipulation of tumor lactic acidosis. We recently reported that it was lactic acidosis but not lactosis that could protect cancer cells against metabolic stress-induced death [17]. Elevating intratumoal pH may convert lactic acidosis to lactosis, induce quick death of cancer cells, hence could reduce CIN.

In conclusion, glucose deprivation with lactic acidosis – two tumor microenvironmental factors – can induce cancer cell CIN potentially via 3 ways, damaging DNA, upregulating mitotic checkpoint genes, and amplifying centrosome. The feature of CIN induced by glucose deprivation with lactic acidosis is similar to that of aneuploid human tumors. We speculate that glucose deprivation and lactic acidosis are potential inducers of human tumor CIN.

## Supporting Information

Figure S1 **One cycle of glucose deprivation with lactic acidosis followed by nutrient restoration exerts significant effect on mitosis of 4T1 cells.** 4T1 cells were cultured in RPMI-1640 medium containing 3 mM glucose with lactic acidosis for 7 days. The cells surviving through glucose deprivation were then cultured in fresh medium for 48 hours for mitotic recovery. Representative photos show the misaligned chromosome at metaphase (the panels on the top), the lagging chromosome at anaphase (the middle panels), and the nucleoplasmic bridge at telophase (the panels at the bottom).
(TIF)

Table S1 **The primers for quantitative real-time PCR of HCT116 cells.**
(DOC)
