# Supplementary figures and images for: Tumor Environmental Factors Glucose Deprivation and Lactic Acidosis Induce Mitotic Chromosomal Instability – An Implication in Aneuploid Human Tumors
Source: PLoS One. 2013 May 10;8(5):e63054. doi: 10.1371/journal.pone.0063054 (PMC3651256; doi:10.1371/journal.pone.0063054)

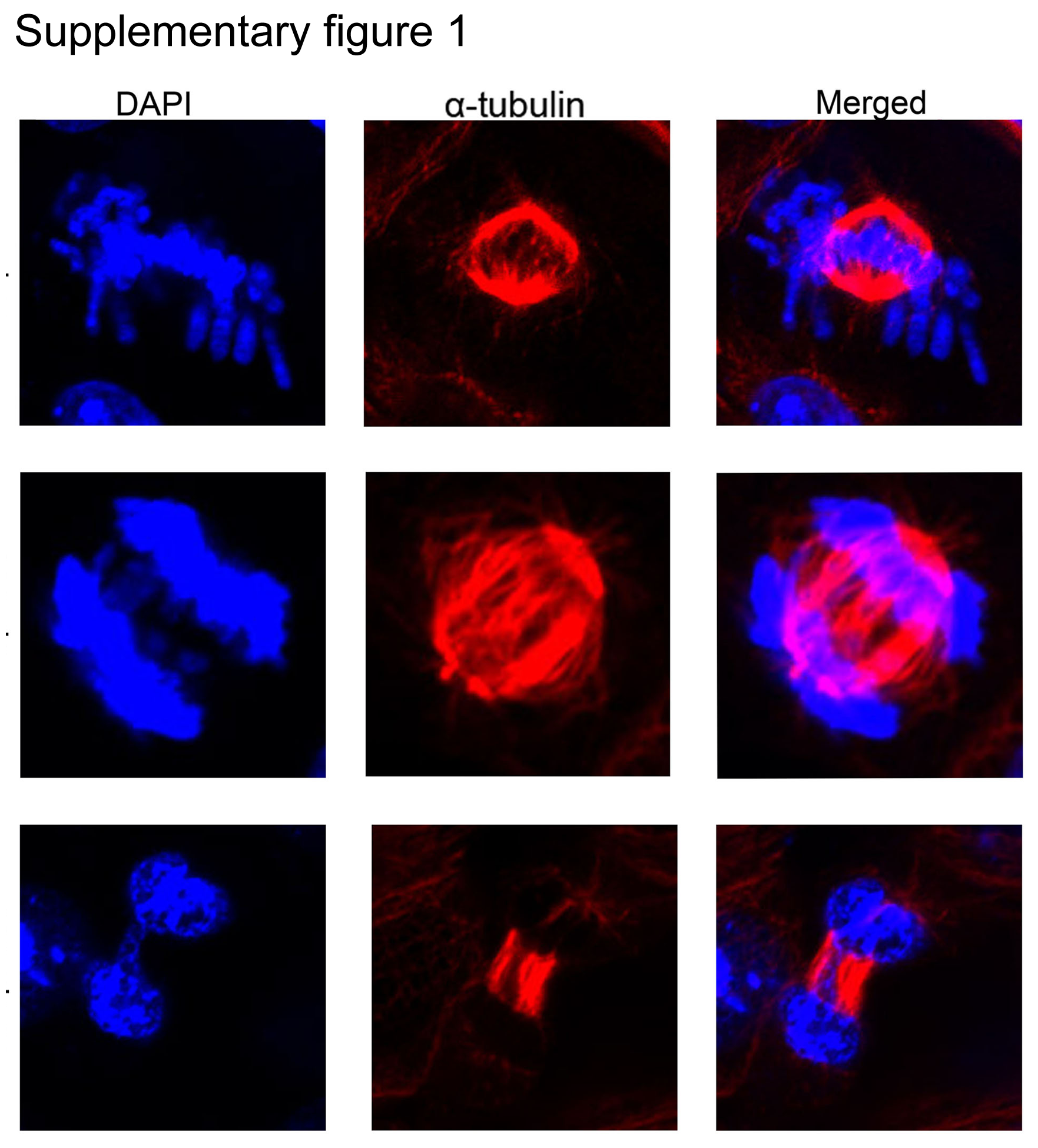

Supplement: Figure S1 — One cycle of glucose deprivation with lactic acidosis followed by nutrient restoration exerts significant effect on mitosis of 4T1 cells. 4T1 cells were cultured in RPMI-1640 medium containing 3 mM glucose with lactic acidosis for 7 days. The cells surviving through glucose deprivation were then cultured in fresh medium for 48 hours for mitotic recovery. Representative photos show the misaligned chromosome at metaphase (the panels on the top), the lagging chromosome at anaphase (the middle panels), and the nucleoplasmic bridge at telophase (the panels at the bottom). (TIF) [file pone.0063054.s001.tif]
