# Supplementary material for: Tumor Environmental Factors Glucose Deprivation and Lactic Acidosis Induce Mitotic Chromosomal Instability – An Implication in Aneuploid Human Tumors
Source: PLoS One. 2013 May 10;8(5):e63054. doi: 10.1371/journal.pone.0063054 (PMC3651256; doi:10.1371/journal.pone.0063054)
Supplement: Table S1 — The primers for quantitative real-time PCR of HCT116 cells. (DOC) [file pone.0063054.s002.doc]

**Table S1.** The primers for quantitative real-time PCR of HCT116 cells.

| Bub1 | GAGTGATATCTTCAGCTTGTG | AACAACCTGCTCAACATCAAC |
| --- | --- | --- |
| Bub1b | ACGTTATTAGAAAGAGCTGTAG | CATATCCAAAGGCTCATTGC |
| Bub3 | ATGCGGCTCAAGTACCAGC | GATCAGTGTTCAAATCATGC |
| Mad2 | GGTCCTGGAAAGATGGCAG | ATCACTGAACGGATTTCATCC |
| Cdc2 | CAAATATAGTCAGTCTTCAGGATG | CCTGTAGGATTTGGTATAAATAAC |
| Cyclin B1 | CAGCTCTTGGGGACATTGGTAACA | ATTGGGCTTGGAGAGGCAGTATCA |
| GAPDH | ACCCACTCCTCCACCTTTGA | CTGTTGCTGTAGCCAAATTCGT |
